# Socio-demographic determinants and prevalence of Tuberculosis knowledge in three slum populations of Uganda

**DOI:** 10.1186/1471-2458-12-536

**Published:** 2012-07-23

**Authors:** Ekwaro A Obuku, Clea Meynell, Jemimah Kiboss-Kyeyune, Simon Blankley, Christine Atuhairwe, Evelyn Nabankema, Morris Lab, Nikki Jeffrey, David Ndungutse

**Affiliations:** 1 International Medical Foundation, P.O Box 8177, Kampala, Uganda; 2 Target TB, Refuge House, 49-50 North Street, Brighton, BN1 1RH, United Kingdom; 3 International Health Sciences University, P. O Box 7782, Kampala, Uganda; 4 Joint Clinical Research Centre, P.O Box 10005, Kampala, Uganda

**Keywords:** TB, Knowledge, Assessment, Slums, Advocacy, Communication and social mobilisation, Uganda

## Abstract

**Background:**

Knowledge of tuberculosis has been shown to influence health seeking behaviour; and urban slum dwellers are at a higher risk of acquiring tuberculosis than the general population. The study aim was to assess knowledge of tuberculosis and identify the associated socio-demographic determinants, in order to inform tailored interventions for advocacy, communication and social mobilisation in three urban-slum communities of Uganda.

**Methods:**

A cross-sectional survey of 1361 adults between April and October 2011. Data was analyzed by descriptive statistics. Adjusted odds ratios (aOR) and 95% confidence intervals (95% CI) of potential determinants of tuberculosis (TB) knowledge were estimated by multivariable ordinal logistic regression using Stata 11.2 software.

**Results:**

We found low knowledge of TB cause (26.7%); symptoms (46.8%), transmission (54.3%), prevention (34%) and free treatment (35%). Knowledge about TB treatment (69.4) and cure (85.1) was relatively high. Independent determinants of poor knowledge of TB in the multivariable analysis included (aOR, 95% CI) lack of formal education (0.56; 0.38 – 0.83, P = 0.004), unemployment (0.67; 0.49 – 0.90, P = 0.010) and never testing for HIV (0.69; 0.51 – 0.92, P < 0.012). Whilst, older age (1.73; 1.30 – 2.29, P < 0.001) and residing in Lira (2.02; 1.50 – 2.72, P < 0.001) were independent determinants of higher knowledge of TB.

**Conclusion:**

This study revealed deficiencies in the public health knowledge about TB symptoms, diagnosis and treatment among urban-slum dwellers in Uganda. Tuberculosis control programmes in similar settings should consider innovative strategies for TB education, advocacy, communication and social mobilisation to reach the youth, unemployed and less-educated; as well as those who have never tested for HIV.

## Background

World over, tuberculosis (TB) is a major cause of illness even though drugs to cure this disease have been available for the past 60 years [1,2]. Uganda is one of the 22 High Burden Countries with an incidence rate of 209 per 100,000 population [1] and a TB Case Detection Rate that has stagnated for the past 5 years, hovering between 37% and 61% [1,3] which signifies a need to go back to the drawing board.

Previous international studies involving different communities including TB patients in Low and Middle Income Countries [4-6] as well as in the developed world [7] suggest that lack of TB knowledge is an important barrier to TB diagnosis and treatment. And that TB knowledge varied by rural-urban setting, gender and education level. In Uganda, one qualitative study focused on people living in rural areas [8] whilst others investigated medical students [9,10] and factors influencing treatment among TB patients [11-15]. To our knowledge no research on this topic has considered the urban slum population in Uganda.

The need to tackle the social determinants of TB has never been more urgent than today [16,17]. TB continues to cluster in disadvantaged groups including urban slum dwellers, who are more-often-than-not the poor and hungry [18-21]. Uganda’s rapid population growth of 3.2% is one of the highest in the world [22], which coupled with migration from the rural areas [23] contributes to the mushrooming of urban slums and consequently a higher burden of TB in these settings. Yet, empowering people with TB and the affected communities is a key pillar of the Stop TB strategy that has not been sufficiently addressed in Uganda *(Personal communication: Dr. Adatu Francis, National TB Programme Manager)*. The study aim was to assess knowledge of tuberculosis and identify the associated socio-demographic determinants, in order to inform tailored interventions for advocacy, communication and social mobilisation in three urban-slum communities of Uganda.

## Methods

### Setting

Three urban centres in Uganda at different levels of development namely Wobulenzi Town Council, Lira Municipal Council, and Makindye Division in Kampala City. Their 2010 projected populations were 23,100; 108,600 and 403,700 respectively. These three areas report a high annual burden of TB and have above–national HIV prevalence estimates [22,24,25].

### Design and sampling procedures

A cross-sectional survey was conducted between April and October 2011 as part of larger study assessing socio-economic vulnerabilities to TB in urban slums. A list of 58 villages was drawn based on the following criteria: settlements that were informal, unplanned, with limited social services, of high population density and low income. From this list, 35 villages (Wobulenzi = 10; Lira = 10; Kampala = 15) to be visited were randomly selected using open source software (*OpenEpi version 2.3.3*) [26]. The Field Officer (FO) liaised with Local Council chairmen to identify the “centre” and boundaries of the village. Once the study team assembled at the “centre”, selection of the first respondent was done by spinning a bottle and following the direction where it pointed. Every third or fifth house was visited depending on the density of settlements to cover all four directions of the village. Respondents, one per house, who were 18 years or older and gave informed consent were eligible. Health workers were excluded.

### Data collection and management

A survey questionnaire was designed to capture data on respondent demographics and TB knowledge with adaptations from the WHO guide on conducting surveys for advocacy-communication-and-social-mobilization (ACSM) for TB control [27]. The tool was translated into local languages (Luganda and Lango) and piloted by a mix of university students and members of the village-health-teams, fluent in both English and the local languages. This team received training in basic interview techniques prior to the study. They were supervised by experienced FOs to assure responsible conduct and data quality. Data was entered into Epi Info^TM^ version 3.5.3 (Centres for Disease Control and Prevention, Atlanta, USA).

### Outcome measures and exposure variables

The main outcome measure was the level of TB knowledge stratified as knowledge of cause, symptoms, transmission, risk groups, prevention, and treatment. Exposure variables included age, sex, marital status, educational level, employment, HIV-testing history, study site, distance from health unit and ownership of a communication device.

### Sample size estimation

A sample size was independently estimated for each study area taking into account a maximum variability of 0.5 in the frequency of TB knowledge, ±0.05 precision, and 95% confidence interval. This yielded 430 participants per study site and 1290 in total after adjusting for a 0.1 proportion of persons not able to be contacted.

### Statistical considerations

Data was analyzed using Intercooled Stata^TM^ version 11.2 (StataCorp, College Station, Texas, USA). Demographic and outcome data was summarized into frequencies, percentages and measures of central tendency. Responses to the 10 questions about TB were scored (one point for correct and zero for incorrect) and categorized into 0 – 25, 26 – 50, 51 – 75 and 76 – 100 percent corresponding to poor, low, moderate and high TB knowledge respectively as a composite outcome. This distinguishes those with almost no knowledge at all (poor) from those with some knowledge about TB (low). The association between exposure variables and TB knowledge was explored by univariate analysis, with crude odds ratios (OR) and 95% confidence intervals. Tests were two-sided and considered significant if *P* < 0.05. Potential determinants of TB knowledge with *P* < 0.25 at univariate level were included in a multivariable ordinal logistic regression model to estimate their adjusted odds ratios (aOR). Multiple imputations by chained equations was employed for sensitivity analysis of the missing data using the Stata command “ice” and “mim”, assuming a Missing At Random mechanism. All missing baseline variables and the outcome, TB knowledge, were included to create five imputed data sets.

### Ethical considerations

This study was approved by the Mulago National Referral and Teaching Hospital Research Ethics Committee (MREC 501) and Uganda National Council for Science and Technology (SS 2571).

## Results

### Socio demographic characteristics

We interviewed 1361 eligible participants; 449 (33%) in Wobulenzi, 470 (34.5%) in Lira and 442 (32.5%) in Makindye. Their median age was 30 years (IQR, 25 – 40). The majority of respondents were female (72.9%); and 65.6% were married or cohabiting. A half (48.9%) had attained secondary school, whilst 11% had never attended formal education. Most were self employed (63.7%) with few having paid employment (18.5%), whilst 78.5% said they had been tested for HIV before. Seventy percent lived within 1 km of a health facility; 70% owned a Radio, 30.7% a TV and 37.4% a mobile phone (Table 1). The proportion of missing data was less than 5% and there were no statistically significant differences in baseline characteristics with the complete cases.

**Table 1:** Socio-demographic characteristics of respondents across the study areas

**Characteristic**	**All Study Areas**	**Wobulenzi**	**Lira**	**Makindye**
**Age**^***Φ***^	**N = 1361 (%)**	**n = 442 (%)**	**n = 470 (%)**	**n = 449 (%)**
Median (IQR)	30 (25 – 40)	32 (25 – 44)	30 (25 – 40)	29 (24 – 36)
18 – 39	987 (73.8)	298 (67.6)	331 (72.6)	358 (81.2)
40 – 59	275 (20.6)	109 (24.7)	98 (21.5)	68 (15.4)
≥ 60	76 (5.7)	34 (7.7)	27 (5.9)	15 (3.4)
**Gender**^***§***^				
Female	995 (72.9)	333 (75.3)	315 (67)	342 (76.3)
Male	370 (27.1)	109 (24.7)	155 (33)	106 (23.7)
**Marital Status**^***§***^				
Married/Cohabiting	892 (65.6)	297 (67)	281 (60)	314 (69.9)
Never Married	245 (18)	64 (14.5)	104 (22.2)	77 (17.2)
Previously Married	223 (16.4)	82 (18.5)	83 (17.7)	58 (12.9)
**Education Level**^***Φ***^				
Secondary & above	664 (48.9)	211 (47.7)	195 (41.6)	258 (57.6)
Primary	545 (40.1)	189 (42.8)	206 (43.9)	150 (33.5)
No School	150 (11)	42 (9.5)	68 (14.5)	40 (8.9)
**Source of Livelihood**^***Φ***^				
Self Employed	862 (63.7)	293 (66.3)	343 (73.4)	226 (50.8)
Employment Income	251 (18.5)	45 (10.2)	77 (16.5)	129 (29)
None	241 (17.8)	104 (23.5)	47 (10.1)	90 (20.2)
**Ever done HIV test**^***Φ***^				
Yes	1053 (78.5)	278 (63.9)	390 (84.2)	385 (86.7)
No	289 (21.5)	157 (36.1)	73 (15.8)	59 (13.3)
**Distance to Health Unit**^***Φ***^				
< 1 km	947 (70)	380 (86.2)	259 (55.6)	308 (69.1)
>1 km	406 (30)	61 (13.8)	207 (44.4)	138 (30.9)
Communication device*				
Radio ^*Φ*^	958 (70.1)	307 (69.3)	380 (80.5)	271 (60.1)
Mobile Phone^*§*^	511 (37.4)	139 (31.4)	192 (40.7)	180 (39.9)
TV ^*Φ*^	419 (30.7)	108 (24.4)	71 (15)	240 (53.2)
Print Media^*§*^	39 (2.9)	4 (0.9)	17 (3.6)	18 (4)

*Multiple responses question.

Differences across the study sites ^§^p < 0.01 &^Φ^ p < 0.001.

^ɱ^ Variables with missing data.

### Awareness of TB disease

Many participants (1139, 83.7%) were aware of TB, initially from some form of contact with a TB patient (51.9%) than from the media (29.3%). Current TB information was commonly received through radio (50%) followed by the formal health system (25.5%). Those who said they had never heard of TB (222, 16.3%) were excluded from further analysis about TB knowledge.

### Knowledge of TB disease

Few respondents identified a germ as the cause of TB (26.7%); with smoking mentioned most commonly (37.1%), whilst a third (27.7%) did not know the cause of TB (Table 2). Coughing was the most frequently mentioned symptom reported as any cough (46.4%) or cough ≥ 2 weeks (46.8%). Half the respondents (54.3%) said TB transmission is airborne. The most frequently mentioned means of TB prevention was avoiding sharing dishes (42.9%), followed by *Cough hygiene* (34%), which was considered the correct response. Two thirds (68.7%) said that although anybody can get TB, persons with HIV/AIDS (19.5%) were at increased risk followed by alcoholics (12.7%), drug users (10.2%) and the poor (5%).

**Table 2:** **Knowledge of Tuberculosis disease among slum dwellers in Uganda**^***§***^

**Knowledge Scale**	**Response**	**N = 1139 (%)**	**Knowledge Scale**	**Response**	**N = 1139 (%)**
**TB Cause**	Smoking	423 (37.1)	**Who can get TB**	**Φ Anybody**	**783 (68.7)**
	**Φ Germ**	**304 (26.7)**		PHAs	223 (19.5)
	Alcohol	227 (19.9)		Alcoholics	145 (12.7)
	Malnutrition	31 (2.7)		Drug Users	116 (10.2)
	Witchcraft	17 (1.5)		Poor	56 (5)
	Other	190 (16.7)		Prison History	17 (1.5)
	Don’t Know	316 (27.7)		Homeless	11 (0.7)
**TB Symptoms**	**Φ Cough > 2 weeks**	**533 (46.8)**		Other persons	89 (7.8)
	Any Cough	530 (46.4)	*TB Cure	**Φ TB is Curable**	**969 (85.1)**
	Weight Loss	380 (33.4)		TB is not Curable	170 (14.9)
	Chest Pain	314 (27.6)	**How is TB Cured**	**Φ Specific Drugs**	**791 (69.4)**
	Coughing up blood	234 (20.5)		Treatment in Community	200 (17.6)
	Fever	185(16.2)		Herbs	38 (3.3)
	Shortness of Breath	171 (15)		Home Rest Alone	29 (2.5)
	Fever > 14 days	106 (9.3)		Prayer	16 (1.4)
	Fatigue	106 (9.3)		Other Cure	23 (2.0)
	Other Symptoms	61 (5.4)		Don’t Know	58 (5.8)
	Don’t Know	124 (10.9)	**Where is TB Cured**	**Φ Government Facilities**	**911 (80)**
**TB Transmission**	**Φ Airborne**	**618 (54.3)**		Private Clinic	167 (14.7)
	Sharing Utensils	519 (45.6)		NGO/Church Facilities	161 (14.1)
	Sharing Meals	226 (19.8)		Traditional Healer	45 (4)
	Shaking Hands	74 (6.5)		Other Cure Place	14 (1.2)
	Touching public items	59 (5.2)	*How Long is Rx	< 1 month	23 (2.0)
	Other routes	102 (8.9)		1 – 3 months	119 (10.4)
	Don’t Know	180 (15.8)		3 - 6 months	178 (15.6)
**TB Prevention**	Avoid Sharing Dishes	489 (42.9)		**Φ 6 - 8 months**	**371 (32.6)**
	**Φ Cough Hygiene**	**387 (34)**		> 8 months	151 (13.3)
	Drugs	281 (24.7)		Don’t Know	297 (26.1)
	Vaccination/BCG	194 (17)	*Cost of TB Rx	**Φ Free of charge**	**399 (35)**
	Avoid Handshake	98 (8.6)		Expensive	367 (32.2)
	Washing Hands	99 (8.7)		Affordable	85 (7.4)
	Good Nutrition	47 (4.1)		Don’t Know	288 (25.3)
	Closing Home Windows	36 (3.2)			
	Prayer	11 (1)			
	Other means	141 (12.4)			
	Don’t Know	167 (14.7)			

Φ *Correct responses in bold*.

*Non-multiple responses question.

^**§**^Individuals (51) missing baseline data <5% in total.

Rx means treatment.

Eighty five percent said TB is curable, using specific drugs (69.4%). Although 80% knew TB treatment can be obtained from government facilities, only a third (35%) were aware of free-of-charge TB treatment. In fact, 32.2% said TB treatment is expensive and a quarter (25.2%) did not know whether TB treatment was provided free or at a fee. Remarkably, one third mentioned the correct 6 – 8 months duration of TB treatment. Correct responses for TB knowledge are marked (**ϕ**) and in bold (Table 2).

### Determinants of TB knowledge score

Age, gender, education level, employment status, HIV testing history, and distance from health unit; study site and ownership of a radio or a mobile phone were significantly associated with TB knowledge at univariate analysis (Table 3). In the multivariable ordinal logistic regression model (category, aOR, 95% CI, P – value) older age (40 – 59 years; 1.73; 1.30 – 2.29, P < 0.001) and residence (Lira Town; 2.02; 1.50 – 2.72, P < 0.001) were associated with higher TB knowledge scores. Whilst, lack of formal education (0.56; 0.38 – 0.83, P = 0.004), unemployment (0.67; 0.49 – 0.90, P = 0.010), never testing for HIV (0.69; 0.51 – 0.92, P < 0.012) remained statistically significant determinants of poor TB knowledge scores (Table 4). In this final model, the assumption of proportionality of odds was not violated when tested using the Brant test, (Chi square = 13.2; p = 0.982).

**Table 3:** **Univariate analysis of predictors of TB knowledge score among slum dwellers in Uganda**^***§***^

**Variable**	**TB Knowledge Score (N = 1139)**				**OR (95% CI)**	**p-value**
	**Poor**	**Low**	**Moderate**	**High**		
**Total Study Population**	145 (12.7)	428 (37.6)	358 (31.4)	208 (18.3)		
**Age**						0.004
18 – 39	113 (75.5)	328 (72.6)	247 (67.6)	141 (73.8)	1	
40 – 59	22 (17.7)	71 (21.5)	83 (24.7)	53 (20.6)	1.57 (1.21 - 2.05)	
≥ 60	9 (7.3)	21 (5.9)	21 (7.7)	11 (5.7)	1.13 (0.71 - 1.80)	
**Gender**						0.017
Female	115 (79.3)	317 (74.1)	246 (68.9)	145 (69.7)	1	
Male	430 (20.7)	111 (25.9)	111 (31.1)	63 (30.1)	1.33(1.05-1.68)	
**Marital Status**						
Married/Cohabiting	95 (66)	287 (67.4)	227 (63.4)	144 (70.2)	1	
Never Married	20 (13.9)	82 (19.3)	64 (17.9)	37 (18)	1.04 (0.79 – 1.38)	
Previously Married	29 (20.1)	57 (13.4)	67 (18.7)	24 (11.7)	0.90 (0.67 – 1.21)	
**Education Level**						0.008
Secondary & above	60 (41.4)	223 (52.2)	181 (50.6)	122 (58.7)	1	
Primary	59 (40.7)	161 (37.7)	137 (38.3)	73 (35.1)	0.82 (0.66 - 1.04)	
Did Not Attend School	26 (17.9)	43 (10.1)	40 (11.2)	13 (6.3)	0.58 (0.40 - 0.83)	
**Source of Livelihood**						<0.001
Self Employed	79 (54.5)	262 (61.5)	232 (65.2)	134 (65.3)	1	
Employment Income	26 (17.9)	77 (18.1)	74 (20.8)	49 (23.9)	1.17 (0.90 - 1.52)	
None	40 (27.6)	87 (20.4)	50 (14)	22 (10.7)	0.61 (0.47 - 0.79)	
**Ever done HIV test**						
Yes	103 (72.5)	335 (78.6)	296 (83.4)	174 (84.9)	1	
No	39 (27.5)	91 (21.4)	59 (16.6)	31 (15.1)	0.64 (0.49 - 0.84)	
**Distance to Health Unit**						0.014
< 1 km	108 (75)	307 (72.4)	244 (68.4)	134 (64.7)	1	
>1 km	36 (25)	117 (27.6)	113 (31.6)	73 (35.3)	1.34 (1.06 - 1.69)	
**Study Site**						<0.0001
Wobulenzi	61 (42.1)	153 (35.8)	104 (29.1)	56 (26.9)	1	
Lira	23 (15.9)	107 (25)	142 (39.7)	91 (43.4)	2.84 (2.22 - 3.64)	
Makindye	61 (42.1)	168 (39.3)	112 (31.3)	61 (29.3)	1.29 (1.01 - 1.65)	
**Communication device**						
Radio (YES)	93 (64.1)	294 (68.7)	269 (75.1)	159 (76.4)	1	
	52 (35.9)	134 (31.3)	89 (24.9)	49 (23.6)	0.68 (0.54 - 0.87)	
TV (NO)	104 (71.7)	282 (65.9)	252 (70.4)	133 (63.9)	1	
	41 (28.3)	146 (34.1)	106 (29.6)	75 (36.1)	1.08 (0.87 - 1.33)	
Mobile Phone (NO)	96 (66.2)	268 (62.6)	224 (62.6)	110 (52.9)	1	
	49 (33.8)	160 (37.4)	134 (37.4)	98 (47.1)	1.29 (1.04 - 1.61)	

OR – Crude Odds Ratio; CI – Confidence Interval; Level of significance p < 0.05.

^**§**^Individuals (51) missing baseline data <5% in total.

**Table 4:** Multivariate ordinal logistic regression analysis of predictors of TB knowledge score among slum dwellers in Uganda

**Variable**	**COMPLETE CASES (n = 1088)**			**IMPUTED (n = 1139)**		
	**aOR**	**95% CI**	**p-value**	**aOR**	**95% CI**	**p-value**
**Age**						
18 – 39	1	-		1	-	
40 – 59	1.73	1.30 – 2.29	<0.001	1.67	1.26 – 2.20	<0.001
≥ 60	1.46	0.89 – 2.40	0.133	1.49	0.91 – 2.44	0.113
**Gender**						
Female	1	-		1	-	
Male	1.06	0.83 – 1.33	0.611	1.06	0.83 – 1.33	0.611
**Education Level**						
Secondary & above	1	-		1	-	
Primary	0.79	0.63 – 1.01	0.065	0.82	0.64 – 1.03	0.090
Did Not Attend School	0.56	0.38 – 0.83	0.004	0.54	0.37 – 0.79	0.001
**Source of Livelihood**						
Self Employed	1	-		1	-	
Employment Income	1.22	0.91 – 1.64	0.211	1.22	0.91 – 1.64	0.174
None	0.67	0.49 – 0.90	0.010	0.65	0.48 – 0.88	0.005
**Ever done HIV test**						
Yes	1	-		1	-	
No	0.69	0.51 – 0.92	0.012	0.67	0.50 – 0.89	0.006
**Distance to Health Unit**						
<1 km	1	-		1	-	
>1 km	1.16	0.90 – 1.49	0.250	1.19	0.93 – 1.51	0.171
**Study Site**						
Wobulenzi	1	-		1	-	
Lira	2.02	1.50 – 2.72	< 0.001	1.95	1.46 – 2.61	<0.001
Makindye	0.93	0.70 – 1.23	0.611	0.92	0.70 – 1.22	0.577
Communication device*						
Radio (YES)	1	-	0.192	1	-	0.125
	0.85	0.66 – 1.09		0.82	0.64 – 1.05	
Mobile Phone (NO)	1	-	0.345	1	-	0.215
	1.12	0.89 – 1.41		1.15	0. 92 – 1.44	

aOR - Odds Ratio adjusted for all variables in the model; CI – Confidence Interval; Level of significance p < 0.05.

Brant test Chi square =13.2; p = 0.982 for complete cases analysis.

Probability > F; < 0.0001 for significant variables in imputed model.

Probability > F; = 0.1768 for non-significant variables in imputed model.

Probability > F; < 0.0001 for all variables in imputed model.

### Missing data

The following baseline variables were missing: age (18), history of testing for HIV (11), distance (7), employment status (7), marital status (6), gender (1) and education level (1) totaling to 51 (4.5%) incomplete cases. The imputed model was not adversely different from the complete cases analysis (Table 4).

## Discussion

The TB incidence and mortality is declining globally except in Sub-Saharan Africa [1]. Reports from the Uganda National TB Control Programme (NTP) show that TB is more common in urban centres. Kampala city is estimated to have less than 10% of Uganda’s population [22] yet accounts for a quarter of the country’s tuberculosis burden [24]. This reflects the importance of socioeconomic determinants of TB [28].

In this study we report sub-optimal knowledge about TB in the general population of slum dwellers in three geographically distinct communities. We also identified that younger age, not having formal education; unemployment and not having tested for HIV were associated with a lower level of TB knowledge (poor), in the multivariable model. In Uganda, health information on TB and HIV/AIDs is disseminated through education talks in schools, places of work, media and through counseling before testing for HIV/AIDs. It is therefore not surprising that those with lack of education, unemployed, and never tested for HIV sero-status were associated with a lower level of TB knowledge. These groups of people are largely outside the formal TB information flow path way. Notably, except for differences in residence, these associations were marginal with adjusted Odds Ratios less than 2; hence we preferred to limit in-depth discussion of cause and effect.

Although TB awareness was high comprehension of the disease cause, symptoms, transmission and prevention was low. Invalid beliefs about the cause of TB are breeding grounds for myths and misconceptions resulting in stigma, social exclusion and can delay health-seeking behaviour in slum communities [29]. Health education efforts should thus demystify the TB disease process.

Smoking was the most mentioned cause of TB, and indeed 14.7% of men were current smokers (data not shown). The prevalence of smoking may even be higher among unemployed youth, which is a likely scenario in Ugandan slums. A recent meta-analysis [30] found an excess risk of TB infection, disease and mortality associated with smoking tobacco. This therefore presents a port of entry for joint TB and tobacco control in the study areas.

We found discordant knowledge between TB transmission and prevention. While the most frequent answer for TB transmission was *“airborne through coughing”* that for prevention was *“not sharing eating utensils”*. Additionally, few participants understood BCG vaccination as an important TB prevention intervention, despite the high immunization coverage in Uganda. These inconsistencies depict gaps that can be addressed by tailored TB sensitization campaigns and health education in facilities.

Chronic cough, the prime symptom for TB, was known to less than half the respondents. Poor knowledge of TB symptoms may translate to delays in seeking care [29]. These results suggest the need for reinforcement of communication about chronic cough as the cardinal public-health TB symptom, which should trigger seeking healthcare from the formal sector by slum dwellers.

Knowledge of TB treatment and cure was relatively high particularly about using specific drugs from government health units. Nonetheless, lack of knowledge that TB services are free is a potential barrier to seeking TB care. The low response about seeking TB treatment from traditional healers contradicts existing reports that 60% of Ugandans first visit local medicine men, before the formal health sector [31]. This finding may be limited to urban settings, or is a consequence of high awareness of TB cure from designated centres; or perhaps is an effect of social desirability bias.

Older respondents had higher TB knowledge scores, particularly the 40 – 59 age category compared to the younger adults. In two previous studies in resource-constrained settings, older age was associated with earlier TB diagnosis health-seeking behavior [29]. It is possible that there was an age cohort effect in the 40 – 59 age group, who could have had more repeated chances of reinforcing exposure to TB information than younger age groups. This implies efforts to educate the slum population about TB should consider messages that reach the younger generation.

Consistent with previous research [29,32], level of education explained disparities in TB knowledge scores. Those who had never attended formal education had poor knowledge of TB. This calls for innovative action to reach those with limited writing and reading skills through TB drama, verbal and pictorial messages as part of a comprehensive ACSM strategy. For example, utilizing sign posts or radio jingles to intensify TB awareness and prevention campaigns in locally spoken languages.

Those without meaningful employment had significantly lower TB knowledge scores in the multivariable regression model. Indeed the interaction between poverty, ignorance and disease cannot be overemphasized making the unemployed a suitable target group for tailored TB health promotion interventions.

HIV testing was associated with a higher TB knowledge score. Since 2005 Uganda’s Ministry of Health aggressively rolled out routine provider initiated HIV counseling & testing [33] which could be responsible for the high proportion of respondents who had tested for HIV in our study. It is likely that persons with HIV testing history had better general health-seeking behavior and exposure to health information. This important finding presents an opportunity for joint action from the HIV side, that is, routinely screen for TB during HIV Counseling and Testing initiatives.

Differences in study site independently predicted knowledge about TB. Compared to Wobulenzi Town and Makindye in Kampala City, respondents in Lira Municipality had higher TB knowledge scores. This could explain the consistent better performance by Lira with above-target Case Detection and Treatment Success Rates over the past 5 years in contrast to Wobulenzi and Kampala [24]. The NTP in Lira consistently received TB-specific support from donor agencies towards strengthening community based TB care by engaging the sub-county health workers of the village-health-team. This further asserts the case for sustained financial support and community mobilisation.

Noteworthy, ownership of a communication device was not associated with TB knowledge in the multivariable model. Seventy percent of the respondents owned a radio, and half reportedly obtained TB information from radio. This may have implications on the nature or intensity of TB of messages being aired on radio, suggesting the current strategy requires re-evaluation. Additionally, up to two-fifths of the respondents owned a mobile cell-phone yet this was not an independent determinant of TB knowledge probably due to under utilization of this service. Good acceptability has been reported on the use of electronic media for health messages [34] and could be brought on board to raise TB knowledge among urban-slum dwellers, since the mobile phone density in Uganda is projected to increase in the near future [35].

This study had limitations. We restricted sampling to extremely poor unplanned settlements which may not be representative of slums. In addition, those who reportedly never heard of TB were excluded from the final analysis. Consequently, knowledge of TB may have been overestimated, thus limiting the genaralisability of these results. However, we found a generally poor comprehension of TB suggesting that the picture could even be worse. We also enrolled more participants than originally anticipated, but decided to take advantage of the larger sample size. Potential biases arising from recall and socio-desirability were minimized by testing and adjusting the study tools a priori. Missing data was explored statistically and did not show significant impact on the overall findings. Finally, being a cross-sectional study the temporal relationship between certain exposures and TB knowledge remain unclear, for example HIV testing history.

## Conclusions

Our survey identified deficiencies and determinants of the public health knowledge about TB, among slum dwellers of Wobulenzi, Lira and Makindye urban centres in Uganda. Lack of awareness and knowledge about TB is a well described barrier to seeking TB diagnostic and treatment services. Innovative TB advocacy, communication and social mobilisation strategies should be considered and target the youth, less educated and unemployed; as well as who have never had an HIV test. Not least, National TB Programmes and the Stop TB partners in similar settings in low and middle income countries should take action towards integrated TB-tobacco and TB/HIV control.

## Abbreviations

ACSM: Advocacy communication and social mobilization; AIDS: Acquired Immunodeficiency Syndrome; aOR: Adjusted Odds Ratio; BCG: Bacille Calmette Guerin; CI: Confidence Interval; FO: Field Officer; HIV: Human Immunodeficiency virus; NTP: National TB Programme; OR: Odds Ratio; TB: Tuberculosis; USA: United States of America; WHO: World Health Organization.

## Competing interests

The authors declare that they have no competing interests.

## Authors’ contributions

Authors contributed to study conception (CM, ML), design and development (EAO, CM, ML, JK, SB, EN, CA, NJ & DN), statistical data analysis (EAO, CA), initial draft manuscript (EAO), interpretation of the results and appraisal of the manuscript (CM, ML, JK, SB, EN, CA, NJ & DN), and approved the final draft, (EAO, CM, ML, JK, SB, EN, CA, NJ & DN).

## Pre-publication history

The pre-publication history for this paper can be accessed here:

http://www.biomedcentral.com/1471-2458/12/536/prepub
